# Supplementary material for: A fault-intrusion-tolerant system and deadline-aware algorithm for scheduling scientific workflow in the cloud
Source: PeerJ Comput Sci. 2021 Nov 2;7:e747. doi: 10.7717/peerj-cs.747 (PMC8576554; doi:10.7717/peerj-cs.747)
Supplement: Supplemental Information 6 [file peerj-cs-07-747-s006.zip › Myproject1.1/docs/org/cloudbus/cloudsim/network/DelayMatrix_Float.html]

DelayMatrix\_Float


JavaScript is disabled on your browser.


- Overview
- Package
- Class
- Use
- Tree
- Deprecated
- Index
- Help

- Prev Class
- Next Class

- Frames
- No Frames

- All Classes

- Summary:
- Nested |
- Field |
- Constr |
- Method

- Detail:
- Field |
- Constr |
- Method


org.cloudbus.cloudsim.network

## Class DelayMatrix\_Float

- java.lang.Object
- - org.cloudbus.cloudsim.network.DelayMatrix\_Float

- ---

    

  ```
  public class DelayMatrix_Float
  extends java.lang.Object
  ```

  This class represents an delay-topology storing every distance between connected nodes

  Since:
  :   CloudSim Toolkit 1.0

- - ### Field Summary

    Fields

    | Modifier and Type | Field and Description |
    | --- | --- |
    | `protected float[][]` | `mDelayMatrix` matrix holding delay information between any two nodes |
    | `protected int` | `mTotalNodeNum` number of nodes in the distance-aware-topology |
  - ### Constructor Summary

    Constructors

    Constructor and Description

    `DelayMatrix_Float(TopologicalGraph graph,
    boolean directed)`

    this constructor creates an correct initialized Float-Delay-Matrix
  - ### Method Summary

    Methods

    | Modifier and Type | Method and Description |
    | --- | --- |
    | `float` | `getDelay(int srcID, int destID)` |
    | `java.lang.String` | `toString()` this method just creates an string-output from the internal structures... |

    - ### Methods inherited from class java.lang.Object

      `clone, equals, finalize, getClass, hashCode, notify, notifyAll, wait, wait, wait`

- - ### Field Detail


    - #### mDelayMatrix

      ```
      protected float[][] mDelayMatrix
      ```

      matrix holding delay information between any two nodes


    - #### mTotalNodeNum

      ```
      protected int mTotalNodeNum
      ```

      number of nodes in the distance-aware-topology
  - ### Constructor Detail


    - #### DelayMatrix\_Float

      ```
      public DelayMatrix_Float(TopologicalGraph graph,
                       boolean directed)
      ```

      this constructor creates an correct initialized Float-Delay-Matrix

      Parameters:
      :   `graph` - the topological graph as source-information
      :   `directed` - true if an directed matrix should be computed, false otherwise
  - ### Method Detail


    - #### getDelay

      ```
      public float getDelay(int srcID,
                   int destID)
      ```

      Parameters:
      :   `srcID` - the id of the source-node
      :   `destID` - the id of the destination-node

      Returns:
      :   the delay-count between the given two nodes


    - #### toString

      ```
      public java.lang.String toString()
      ```

      this method just creates an string-output from the internal structures... eg. printsout the
      delay-matrix...

      **Overrides:**
      :   `toString` in class `java.lang.Object`


- Overview
- Package
- Class
- Use
- Tree
- Deprecated
- Index
- Help

- Prev Class
- Next Class

- Frames
- No Frames

- All Classes

- Summary:
- Nested |
- Field |
- Constr |
- Method

- Detail:
- Field |
- Constr |
- Method
